# An ultrasonographic analysis of the activation patterns of abdominal muscles in children with spastic type cerebral palsy and in typically developing individuals: a comparative study

**DOI:** 10.1186/s40945-018-0048-x

**Published:** 2018-06-05

**Authors:** Saviour Kweku Adjenti, Graham Jacob Louw, Jennifer Jelsma, Marianne Unger

**Affiliations:** 1 Department of Anatomy, School of Biomedical & Allied Health Sciences, College of Health Sciences, Korle-Bu Campus, University of Ghana, P.O. Box KB 143, Korle-Bu, Accra, Ghana; 2 Division of Clinical Anatomy & Biological Anthropology, Department of Human Biology, Faculty of Health Sciences, University of Cape Town, Cape Town, South Africa; 3 Division of Physiotherapy, Department of Health & Rehabilitation Sciences, Faculty of Health Sciences, University of Cape Town, Cape Town, South Africa; 4 Division of Physiotherapy, Faculty of Medicine & Health Sciences, Stellenbosch University, Stellenbosch, South Africa

**Keywords:** Spastic type cerebral palsy (STCP), Abdominal muscles, Muscle thickness, Utra-sound imaging, Rehabilitation

## Abstract

**Background:**

Abdominal muscles have stiffer appearance in individuals with spastic type cerebral palsy (STCP) than in their typically developing (TD) peers. This apparent stiffness has been implicated in pelvic instability, mal-rotation, poor gait and locomotion. This study was aimed at investigating whether abdominal muscles activation patterns from rest to activity differ in the two groups.

**Method:**

From ultrasound images, abdominal muscles thickness during the resting and active stages was measured in 63 STCP and 82 TD children. The thickness at each stage and the change in thickness from rest to activity were compared between the two groups.

**Results:**

Rectus abdominis (RA) muscle was the thickest muscle at rest as well as in active stage in both groups. At rest, all muscles were significantly thicker in the STCP children (*p* <  0.001). From rest to active stages muscle thickness significantly increased (p <  0.001) in the TD group and significantly decreased (p <  0.001) in the STCP children, except for RA, which became thicker during activity in both groups. In active stages, no significant differences in the thickness in the four abdominal muscles were found between the STCP and the TD children.

**Conclusion:**

Apart from the RA muscle, the activation pattern of abdominal muscles in individuals with STCP differs from that of TD individuals. Further studies required for understanding the activation patterns of abdominal muscles prior to any physical fitness programmes aimed at improving the quality of life in individuals with STCP.

**Trial registration:**

HREC REF: 490/2011. Human Research Ethics Committee, Faculty of Health Sciences, University of Cape Town, South Africa. November 17, 2011.

## Background

The abdominal muscles play an important role in stabilising the trunk and providing postural stability [1]. These muscles include the internal and external oblique muscles (IO and EO respectively), the transverse abdominis (TA) and the rectus abdominis (RA). In children with spastic type cerebral palsy (STCP), poor postural control is noted to be a primary manifestation of the motor dysfunction [2]. The need to target the control of the trunk in therapy as early as possible in children with neurodevelopmental problems has been emphasised by Burtner and co-workers [3]. These authors reported that skeletal muscles possess remarkable plasticity and can quickly gain or lose contractile material according to changes in loading regimens. Therapists often focus, either directly [4] or indirectly [5], on the abdominal muscles for improving postural control and function. Targeting the trunk is particularly common in those children who display an anterior pelvic tilt [4, 6]. This position places a prolonged stretch on the TrA and RA muscles needed to maintain a neutral pelvis and subsequently causes inhibition of the stretch reflex [7] thereby decreasing reactivity in these muscles. Despite the recognised clinical importance of these muscles, it is evident from the literature that little is known about the structure, function and neuronal activity in persons with STCP [8].

Although STCP is a non-progressive disorder, over time secondary complications occur due to weakness and tone imbalance [9]. According to Hungerford and co-workers, the abnormal forces imposed by the muscles on the skeletal system result in biomechanical mal-alignment such as the anterior pelvic tilt mentioned above [10]. Additionally, in individuals with STCP abnormal recruitment such as the top-down recruitment of the trunk muscles is common [11]. This abnormal recruitment has been associated with co-contraction of the extremity muscles [7]. Other abnormalities noted include a method of fixation of the trunk in some STCP population as well as a negligible muscular activity in others [10, 12]. With regards to individuals with STCP, the muscle groups which appear to contribute to this fixation include the flexors, adductors and the internal rotators of the hip, which gives rise to the typical postural and gait patterns seen – couch gait in diplegia and equines gait in hemiplegia [11].

The force-generating capacity of a skeletal muscle and consequently muscle strength is reported to be a composite function of different aspects of the muscle architecture, including thickness [13]. Ultrasound imaging is a non-invasive method of recording changes in muscle thickness during activation, which was first exploited in muscle activity of the myocardium [14]. Ultrasonography has since been used to quantify muscle thickness in individuals with STCP [15, 16].Ultrasound imaging method has been reported to be fast, inexpensive and above all reliable. The advantages of the use of ultrasonography over electromyography technique have been documented by Ohata et al. [16, 17].

The present study aimed to contribute to the understanding of the functioning of abdominal muscles as a group and/or separate muscles of the anterior abdominal wall in individuals with STCP. The specific objectives of the study were to: (i) measure the thickness levels in each of the four anterior abdominal muscles during the resting and active stages and (ii) compare the changes in thickness, herein referred to as activation pattern between these two stages in the two groups. It is expected that the muscles of TD individuals would demonstrate greater activation pattern evidenced by a larger change in thickness from the resting to active stages than their age-matched peers with STCP.

## Methods

The design of this study was descriptive and analytical. Ethical approval was obtained from the Human Research Ethics Committee of the **XXXXXXXXXXXXX** (HREC REF: 490/2011).

### Participants

The STCP group was recruited from individuals attending special schools in **XXXXXXXX** while the TD group comprises children attending mainstream schools in the vicinity of the special schools. Informed consent and or assent were obtained from individuals and or guardians from these convenient sampling. A learner was excluded if he or she had any surgical operation involving the anterior abdominal wall in the last six months before the start of the study.

For the STCP group, the Gross Motor Function Classification Scale [GMFCS] [18] was also used by a neurodevelopmental therapist to determine the level of function of the participants to be included. Only children in levels I-IV formed part of the inclusion criteria. Children at level V were excluded because they were unable to perform the test manoeuvres. Another exclusion criterion for individuals with STCP was an involvement with any medical treatment that would have impacted on muscle function (e.g., Botulinum toxin injection, casting, and surgical intervention such as dorsal rhizotomy and baclofen pump placement) less than six months before the study.

### Assessment

In all participants, anthropometric parameters (i.e. height and weight were measured before ultrasonographic assessment.

A SIEMENS® ACUSONIC X150 ultrasound imaging machine (Munich, Germany) was used to capture the thickness of the four abdominal muscles, rectus abdominis (RA), internal oblique (IO), external oblique (EO) and transverse abdominis (TrA), in both the resting and active stages. To test the muscles in the resting stage, children were asked to lie supine on the plinth with no activity. For the active stage, children were asked to lie supine on the plinth and then asked to perform the following activities: (i) To fully abduct the shoulder joint (ii) to tuck in the chin and lift head and neck slightly towards the chest; and (iii) to flex the hip as far as possible. The performance of these activities was aimed at initiating a simultaneous contraction of the abdominal muscles, which was then measured. The average of these three manoeuvres was recorded as the active stage thickness. The side of active upper or lower limb motion and of abdominal muscle thickness measurement was the affected side in hemiplegic children, the right side in diplegic, quadriplegic and TD children. The principal investigator handled the transducer head (ultra-sound probe) while one of the research assistants, a neurodevelopmental therapist, issued the instructions to the participants.

Using the umbilicus as a landmark the ultrasound probe was placed two to three centimetres from the midline and then was panned around in a semi-circular fashion until the bulk of the image from the deepest lying abdominal muscle, TrA, was observed on the image screen. This position was marked on the skin with a marker pen in order to ensure that the probe was kept in this position for subsequent measurements. The scanning head of the probe was then oriented along the mid-sagittal axis of each of the rest of the three anterolateral abdominal muscle (EO, IO and TrA) in a somewhat oblique fashion. The pressure of the transducer was kept to a minimum by using a generous amount of the contact gel in order to obtain optimum values for muscle thickness. All sites along a muscle from which images were taken at rest were then repeated during each child’s head and shoulder/leg lift movement (active stage). Images were stored on a personal computer and then analysed with ImageJ Microsoft version 1.46, 2011 edition (Richmond, Virginia, USA).

**Fig. 1:**
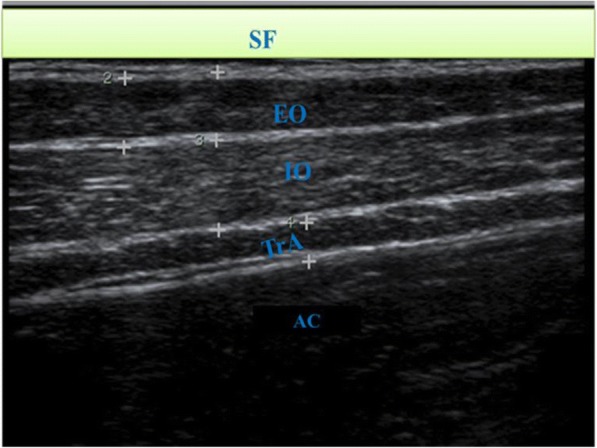
Sonogram showing the three anterolateral muscles. SF = skin and superficial fascia, AC = abdominal cavity. The RA is out of view

*Muscle thickness* (MT) was determined using an electronic calliper on a frozen image. The length of a perpendicular line drawn between the echoes parallel to the fascicles from the deep up to the superficial aponeurosis (inter-fascial planes) was measured (Fig. 1). Since thickness varies along the length of a muscle, measurements were taken at three different points for a particular muscle according to the clarity of the image and the average was recorded for that individual.

Before the test, the intra-rater reliability of the ultrasound measurements was assessed on fifteen (15) randomly selected participants in the STCP and TD groups. This test was performed by the principal investigator (SKA) on different occasions. Measurements were taken at three different points for a particular muscle per participant and the averages were recorded. In each participant, muscle thickness was measured as described above, in both the resting and active stages by SKA. The same assessor repeated the measurements one week later. The outcome of this intra-rater reliability test was expressed as reliability indexes (Intraclass Correlation Coefficient, typical error and mean differences ± SD between the measures taken by the two assessments), showing good to excellent correlation (ICC ≥ 0.80) see Tables 4 and 5 in Appendix).

### Statistical analysis

STATISTICA software package, version 11 (2012) was used to analyse the data. The BMI was calculated using the standard formula, mass (kg)/height (m) x height (m) [19]. Descriptive statistics were presented for the data sets: height, weight, age and muscle thickness. Due to the relatively large sample size, normality was assumed and parametric tests were used for all analyses. The Chi-Square test was used to compare the sex distribution between the STCP and TD groups. Independent t-test was used to statistically compare the means of the two groups in both resting and active stages. A two-way ANOVA with repeated measures was used to determine if there was a significant group-stage interaction that affected muscle thickness. The changes in muscle thickness from rest to the active stage in each group were compared using paired t-test. A 95% confidence interval was used to determine the precision of the estimates of the differences in muscle thickness between the resting and active stages for both groups. Association between muscle thickness and age of all participants was assessed using the Pearson’s correlation coefficient. The level of significance for all statistical tests was set at 0.05.

## Results

Over 200 participants (more than 100 in each group) met the inclusion criteria and were invited to participate. The parents of 145 (63 children with STCP and 82 TD children) gave consent and their children were recruited. The demographic data of all the participants are shown in Table 1. There were no significant differences in age (*p* = 0.102) and in gender (*X*^*2*^ = 0.139; *p* = 0.709) between the two groups (STCP group: mean age 11.2 ± 2.9 years, 55.6% males: TD group: mean age 11.3 ± 2.9 years, 52.4% males). The groups were also similar as regards height and weight. `However, the children with STCP were both shorter and heavier than the children in the TD group, and therefore they had a significantly greater BMI (*p* <  0.001).

**Table 1:** Comparison of demographic data between the groups

	STCP		TD		t-value	Df	*p*-value
	Mean	SD	Mean	SD			
Age (years)	11.89	2.92	11.05	2.92	1.65	143	0.102
Height (cm)	139.19	16.04	143.32	17.13	−1.48	143	0.142
Weight (kg)	39.68	10.28	38.75	12.38	0.48	143	0.629
BMI (kg.m^−2^)	20.14	2.16	18.37	2.62	4.34	143	< 0.001

The distribution of the various subtypes of the spastic cerebral palsy is shown in Table 2. More than half (*N* = 34) of the STCP participants were able to move independently without appliances (GMFCS level I). There were between eight and eleven children in each of the other levels. Forty-four of the participants had hemiplegia (Table 2).

**Table 2:** Gross Motor Classification System Level per distribution of STCP (*N* = 63)

LEVEL	Hemiplegia	Diplegia	Quadriplegia	Total for level
I	29	5	0	34
II	9	2	0	11
III	3	4	1	8
IV	3	3	4	10
All Groups	44	14	5	63

Note the high distribution of the hemiplegic subtypes of STCP in this study

Note also that only four disability levels (ambulatory individuals) were recruited

**Fig. 2:**
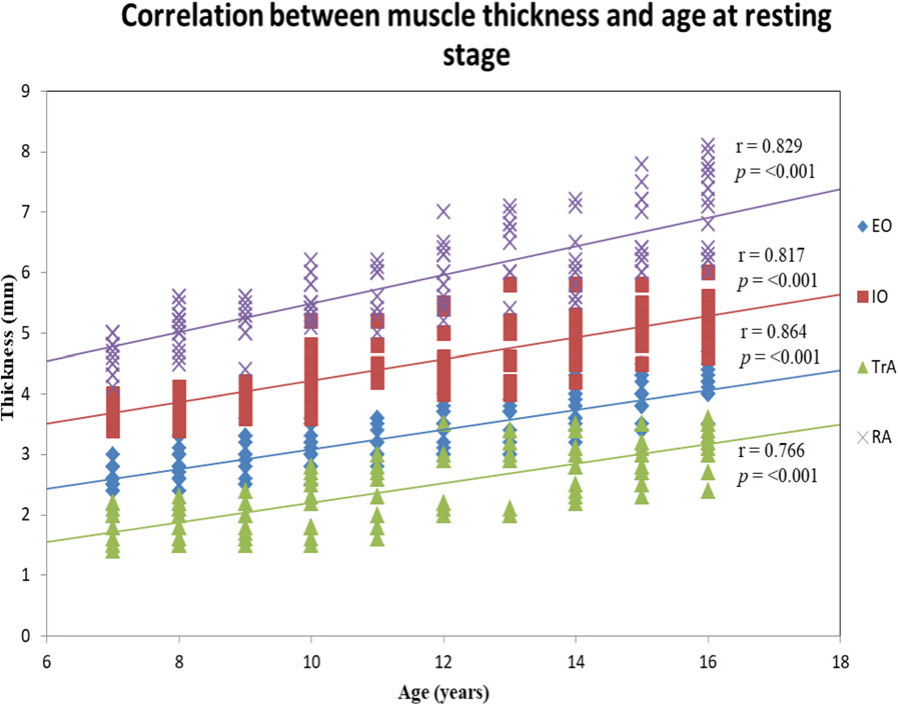
Scatterplot diagrams of age of participants and mean resting muscle thickness for both groups combined (*N* = 145), showing Pearson’s correlation (r) between muscles thickness and age. All correlation coefficients were significant at *p* <  0.001. EO = external oblique; IO = external oblique; TrA = transversus abdominis; RA = rectus abdominis

Muscle thickness at rest showed a significant positive association with the age of participants (*r* = 0.766–0.864, *p* <  0.001) in both groups (Fig. 2). The RA muscle remained the thickest muscle across all ages in both groups, followed by the IO, EO and TrA muscles.

**Fig. 3:**
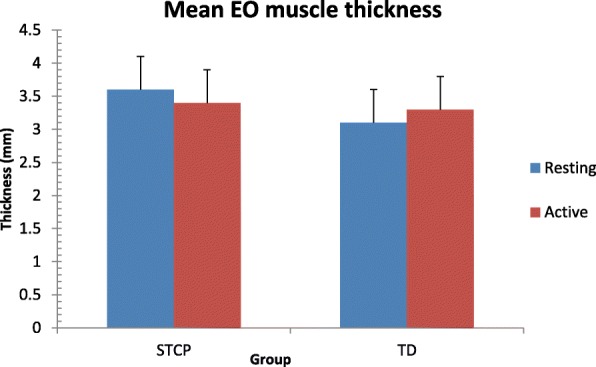
Error bar plots showing the mean thickness for the external oblique muscle (EO) during resting and active stages in both groups. STCP = spastic type cerebral palsy; TD = typically developing

**Fig. 4:**
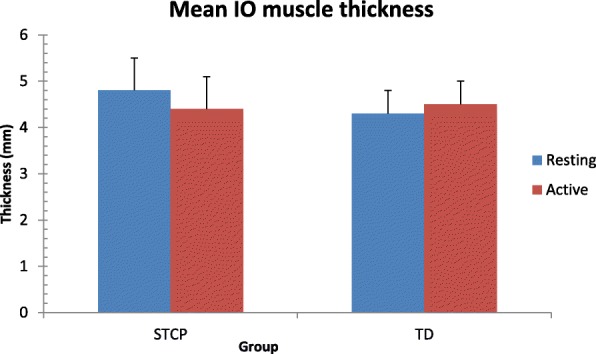
Error bar plots showing the mean thickness for the internal oblique muscle (IO) during resting and active stages in both groups. STCP = spastic type cerebral palsy; TD = typically developing

**Fig. 5:**
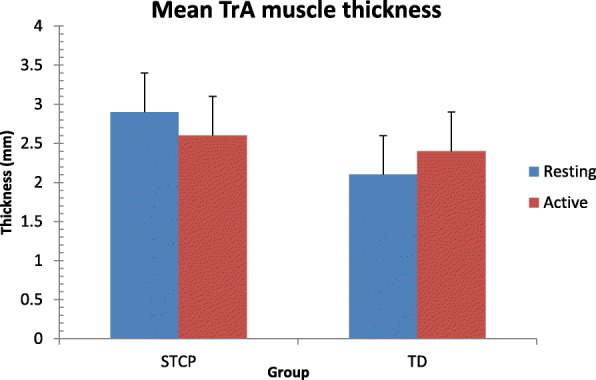
Error bar plots showing the mean thickness for the transverse abdominis muscle (TrA) during resting and active stages in both groups. STCP = spastic type cerebral palsy; TD = typically developing

**Fig. 6:**
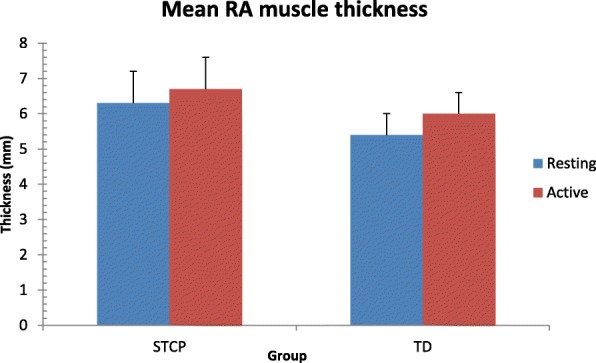
Error bar plots showing the mean thickness for the rectus abdominis muscle (RA) during resting and active stages in both groups. STCP = spastic type cerebral palsy; TD = typically developing

The average thickness of each muscle at rest and the active stages in the two groups and the results of paired and unpaired t-tests are reported in Table 3. Data are also shown graphically in Figs. 3, 4, 5 and 6. At rest, all muscles were significantly thicker in the STCP than in the TD children (*p* <  0.001). From rest to active stages, muscle thickness significantly increased (*p* <  0.001) in the TD group and significantly decreased (*p* <  0.001) in the STCP children, except for RA, which became thicker during activity in both groups. The repeated measures ANOVA confirmed the signicant (*p* <  0.001) group x stage interaction effect on thickness for all muscles (EO: F (1,143) = 283.097; IO: 310.669; TrA: 601.925; RA: 25.278). In active stages, no significant difference in the thickness of the four abdominal muscles was found between the STCP and TD children, and the RA muscle was still the thickest muscle in both groups.

**Table 3:** Comparison of average raw muscle thickness at rest and active stage in both STCP and TD groups

		Rest	Activity	Mean diff.	*T*-statistic	p-value	95% CI of diff.
EO	STCP	3.64 ± 0.50	3.36 ± 0.52	0.28	12.43	< 0.001	0.24 to 0.32
	TD	3.08 ± 0.50	3.29 ± 0.50	−0.21	−11.21	< 0.001	−0.24 to − 0.18
	Mean diff.	0.56	0.08				
	95% CI of diff.	0.47 to 0.65	0.00 to 0.16				
	*p*-value	< 0.001	0.086				
IO	STCP	4.76 ± 0.69	4.43 ± 0.73	0.33	11.04	< 0.001	0.27 to 0.38
	TD	4.25 ± 0.52	4.45 ± 0.52	−0.20	−15.35	< 0.001	−0.23 to −0.17
	Mean diff.	0.51	−0.02				
	95% CI of diff.	0.40 to 0.62	−0.12 to 0.08				
	*p*-value	< 0.001	0.104				
TrA	STCP	2.86 ± 0.49	2.56 ± 0.48	0.30	16.30	< 0.001	0.27 to 0.33
	TD	2.10 ± 0.53	2.38 ± 0.49	−0.28	−18.49	< 0.001	−0.31 to − 0.25
	Mean diff.	0.76	0.18				
	95% CI of diff.	0.66 to 0.86	0.10 to 0.26				
	*p*-value	< 0.001	0.082				
RA	STCP	6.33 ± 0.91	6.70 ± 0.93	−0.37	−17.65	< 0.001	−0.41 to −0.33
	TD	5.44 ± 0.59	5.97 ± 0.63	−0.53	−23.97	< 0.001	−0.57 to − 0.49
	Mean diff.	0.89	0.73				
	95% CI of diff.	0.75 to 1.03	0.59 to 0.87				
	p-value	< 0.001	0.130				

*EO* external oblique muscle, *IO* internal oblique muscle, *TrA* transverse abdominis muscle, *RA* rectus abdominis muscle, *STCP* spastic type cerebral palsy, *TD* typically developing developing, *diff*. difference

## Discussion

The counterintuitive results that emerged from the study were that apart from the RA, the muscles of the STCP were thicker at rest, than the muscles of the TD children and then decreased in thickness when the active manoeuvres were performed. The RA muscle of the STCP group resembled those of TD children in both thickness and activation patterns. It may be that the abdominal muscles have to stabilise the multi-jointed spine and pelvis even in supine position, which requires both concentric and eccentric contraction, compared to the comparatively simple task of controlling one or two joints in the case of the lower limb muscles. Therefore, since individuals with STCP require more support of the trunk than their TD counterparts, the trunk stabilising role could inevitably predispose the abdominal muscles in individuals with STCP to become relatively thicker at rest than for the TD group. Ohata and co-workers also found the abdominal muscles thickness in individuals with STCP to be remarkably high at rest [20]. Apart from the work of Ohata and co-workers [20], no other comparable results for the abdominal muscles in STCP children are found in the literature.

The size of the RA, the thickest muscle in both groups, may be explained in terms of postural roles. Although recruitment patterns differ between individuals and are influenced by changes in body position, a thicker RA than the rest of the abdominal muscles could probably be a result of the use this muscle in all global movements of the trunk during activity of daily living [21]. It has been suggested that the abdominal muscles may be in constant state of contraction, either due to neurological damage leading to spasticity/hypertonia or to the need to stabilise the trunk in the presence of deficient postural responses [2]. A related study which focused on the neuromuscular activity of the abdominal muscles, reported high EMG activation patterns during periods of inactivity (resting stage) in a cohort of children and adolescents with STCP [2]. Such evidence would support the result from the present study and suggests that the levels of abdominal muscles thickness at rest may have underlying physiological/neurological activity, which needs further investigation. This physiological/neurological activity would also partially explain why the muscles in the STCP group showed less change during neck and lower/upper limb activity, as they might have been in a state of contraction formerly at rest. However, with the exception of RA, muscle thickness in the STCP group was less when active than during the resting stage, indicating that these other abdominal muscles might differ structurally and functionally from those of TD individuals. Although similar decrease in abdominal muscle thickness from resting to active stage was reported by Ohata et al. [20], further investigations would be required to fully understand the anatomy and physiology of these muscles in individuals with STCP.

In both groups, the level of thickness and activation pattern of the RA muscle suggest that this muscle may be suitable to take on the stabilising and flexing role of the other abdominal muscles on activity. In individuals with STCP, the activation pattern of the RA muscle might result from the inhibition of the other abdominal muscles that decreased their level of contraction during activity. From a functional perspective, weak and inadequately contracting oblique muscles are often associated with a lack of trunk rotation and an accompanying altered gait, all features that characterise individuals with STCP. It could therefore, be inferred from the results of this study that the function of trunk rotation by the EO and IO muscles in individuals with STCP could be sacrificed above the need to stabilise the trunk. The latter role taken on almost exclusively by the RA muscle: a trunk flexor with no rotatory moment on activity [22].

The larger thickness found at rest in children with STCP in comparison with their TD counterparts might potentially indicate muscle hypertrophy. In that case, however, except for the RA muscle, children with STCP seem lack the ability to activate the contractile materials optimally as a decrease in thickness from resting to active stages was observed. Alternatively, a varying amount of non-contractile materials in these muscles might have contributed to the differences in thickness between the two groups. An inclusion of this aspect in further investigation would be useful for a better understanding.

This study has some limitations. First more than two-third (^2^/_3_) of the children with STCP enrolled were hemiplegic and more than half were at the highest functional level. These proportions limit the generalisation of our finding since they are higher than those in epidemiological studies [23]. This discrepancy may be due to the sampling from special schools in which only educable children are admitted and more severe disability levels, which are associated with severe mental involvement [24], are excluded. Moreover, the researcher who analysed the images was not blinded and this may have introduced some bias.

Finally, in the children with STCP, the chosen neutral (plinth) position might not reflect the true thickness of abdominal muscles at rest. Actually, in children with some neuromuscular deficit the abdominal muscles might have been conditioned to contract also in that position in an attempt to stabilise the spine and pelvis.

## Conclusion

The resting stage thickness of the anterior abdominal wall muscles of individuals with STCP is greater than those of their TD counterparts. The change in muscle thickness of abdominal muscles from the resting to active stages in individuals with STCP differs from that of TD children except for the RA muscle, thereby implying that the RA muscle is unaffected or less affected by the condition. This knowledge could be useful in the problem-solving approaches with regard to the functional aspect of the musculoskeletal system in the provision of quality-of-life benefits for individuals with STCP. Further research is needed to examine the patterns of abdominal muscle activity through dynamic electromyography (EMG).

## Highlights of this study include

- In children with STCP, the activation patterns of abdominal muscles, except for the RA differ from those found in TD children.
- Apart from the RA, the rest of the abdominal muscles in children with STCP are thicker at rest than during the active stage which might indicate hypertonicity or increased need to stabilise the trunk.
- The role of abdominal muscles in stabilising the trunk / pelvis in individuals with STCP requires further investigation.

## Appendix

**Table 4:** Intra-rater reliability of the ultrasound measurements (direct method) in the feasibility study for the STCP group (*n* = 15). Data were expressed as typical error and intra-class coefficients (ICC) with their 95% confidence intervals (CI) and mean differences ± standard deviation (SD)

Muscle & Stage	Typical Error 95% CI	ICC 95% CI	Mean diff ± SD
EO R	0.65 (0.06–0.24)	0.81 (0.66–0.96)	− 0.19 ± 0.04
EO Ac	0.59 (0.07–0.18)	0.88 (0.76–0.97)	−0.10 ± 0.04
IO R	0.60 (0.06–0.20)	0.82 (0.86–0.98)	0.10 ± 0.08
IO Ac	0.62 (0.08–0.24)	0.80 (0.65–0.95)	0.11 ± 0.05
TrA R	0.64 (0.05–0.20)	0.82 (0.68–0.98)	0.17 ± 0.07
TrA Ac	0.63 (0.06–0.22)	0.85 (0.70–0.94)	0.08 ± 0.06
RA R	0.60 (0.08–0.25)	0.86 (0.75–0.98)	−0.11 ± 0.06
RA Ac	0.62 (0.07–0.20)	0.88 (0.76–0.97)	−0.19 ± 0.03

Key: *EO R* external oblique resting stage, *EO Ac* external oblique active stage, *IO R* internal oblique resting stage, *IO Ac* internal oblique active stage, *TrA R* transverse abdominis resting. *TrA Ac* transverse abdominis active stage, *RA R* rectus abdominis resting stage, *RA Ac* rectus abdominis active stage

**Table 5:** Intra-rater reliability of the ultrasound measurements (direct method) in the feasibility study for the TD group (*n* = 15). Data were expressed as typical error and intra-class coefficients (ICC) with their 95% confidence intervals (CI) and mean differences ± standard deviation (SD)

Muscle & Stage	Typical Error 95% CI	ICC 95% CI	Mean diff ± SD
EO R	0.62 (0.06–0.22)	0.80 (0.66–0.96)	−0.13 ± 0.08
EO Ac	0.60 (0.08–0.20)	0.82 (0.76–0.97)	−0.02 ± 0.09
IO R	0.64 (0.05–0.22)	0.81 (0.86–0.98	−0.16 ± 0.06
IO Ac	0.62 (0.06–0.21)	0.79 (0.65–0.95)	−0.09 ± 0.08
TrA R	0.64 (0.04–0.20)	0.84 (0.68–0.98)	0.09 ± 0.09
TrA Ac	0.64 (0.06–0.22)	0.85 (0.72–0.94)	0.09 ± 0.09
RA R	0.66 (0.08–0.24)	0.85 (0.75–0.98)	−0.10 ± 0.09
RA Ac	0.63 (0.06–0.20)	0.86 (0.76–0.96)	−0.01 ± 0.03

Key: *EO R* external oblique resting stage, *EO Ac* external oblique active stage, *IO R* internal oblique resting stage, *IO Ac* internal oblique active stage, *TrA R* transverse abdominis resting. *TrA Ac* transverse abdominis active stage, *RA R* rectus abdominis resting stage, *RA Ac* rectus abdominis active stage

## Abbreviations

BMI: Body mass index
CP: Cerebral palsy
EMG: Electromyograph
EO: External oblique muscle
GMFCS: Gross motor function classification system
IO: Internal oblique muscle
RA: Rectus abdominis muscle
sEMG: Surface electromyography
STCP: Spastic type cerebral palsy
TD: Typically developing
TrA: Transversus abdominis muscle
